# The mediating effect of geospatial thinking on the relationship between family capital and sense of place

**DOI:** 10.3389/fpsyg.2022.918326

**Published:** 2022-10-26

**Authors:** Jianzhen Zhang, Xiaoyu Liang, Ting Su, Xinyao Li, Jiahao Ge, Zhenni An, Yanhua Xu

**Affiliations:** College of Geography and Environmental Science, Zhejiang Normal University, Jinhua, China

**Keywords:** family capital, sense of place, geospatial thinking, mediating and buffering effects, upper-secondary-school students

## Abstract

Few studies have examined how family capital affects the sense of place, and the effect of spatial thinking on the relationship between the two is unclear. This study constructs a mediation model to examine the impact of family capital on sense of place and the mediation effect of geospatial thinking. A total of 1,004 upper-secondary-school students were surveyed using the Family Capital Questionnaire, the Geospatial Thinking Test, and the Sense of Place Scale. The correlation analysis showed that family capital has a positive effect on both sense of place and geospatial thinking. Moreover, there is also a significant positive correlation between geospatial thinking and sense of place. The results of mediation analysis indicated that geospatial thinking plays mediating and buffering roles in the relationship between family capital and sense of place after controlling for gender and residential address. The direct and indirect effects accounted for 73.31 and 26.69% of the total effect, respectively. Specifically, family capital is a significant positive predictor of both sense of place and geospatial thinking, and geospatial thinking partially mediates the relationship between family capital and sense of place. Students from better family backgrounds are more likely to have a better geospatial thinking and sense of place, as well as geospatial thinking promotes the development of a sense of place. Therefore, both family capital and geospatial thinking should be considered when we want to examine and develop individuals’ level of sense of place.

## Introduction

In recent years, sense of place has gradually become a research priority (Procentese and Gatti, 2019; Bissell, 2021). Numerous studies have shown that a sense of place satisfies people’s need for attachment and belonging to a place. Derrien and Stokowski (2014) found that sense of place can improve quality of life. In addition, it can promote pro-environmental behavior, creativity, and academic achievement in geography (Halpenny, 2010; Zhang et al., 2022). Moreover, citizens’ worldview and consumerism were also related to sense of place (Ontong, 2018; Rieh, 2020). In other words, a person’s sense of place affects their ways of thinking, lifestyle, and physical and mental health (Lengen and Kistemann, 2012).

Many factors influence sense of place. Personal factors include gender, age, social status, education level, and length of residence (Relph, 1976). Environmental factors include those related to social environment [social relations, socioeconomic status, religious beliefs, and participation in activities (Williams and Kitchen, 2012)] and to physical environment [local characteristics of the place, natural environment, building facilities, etc. (Stedman, 2002)].

Home is an important environment for understanding spatial (actual and perceived) influences, so the perception of home is an important element of sense of place research (Maxwell, 2003). Families play a key role in people’s health, perceptions, and experiences (Evans and English, 2002; Ackerman et al., 2004; Matthews and Gallo, 2011). Ishizawa (2014) suggested that the higher the education level of a family is, the more it can encourage family members to increase their interaction and connection with the local community. Moskal (2014) found that when the cultural capital of an individual and their family is affirmed in social interactions, that person is better able to integrate into a new environment. In view of the critical role of home on family members’ environmental perceptions and emotional experiences (Thornock et al., 2019), it is crucial to understand how family influences the formation of a sense of place.

Researchers have identified geospatial thinking as the basis of sense of place (Gersmehl and Gersmehl, 2007; Bodzin et al., 2014). Research has confirmed that the human brain is capable of processing spatial information and forming spatial cognition, which can contribute to the development of a sense of place (Lengen and Kistemann, 2012). Related studies have demonstrated a possible relationship between family capital, sense of place, and geospatial thinking. According to the family investment theory of Conger and Donnellan (2007), individuals’ perceptions and their interactions with the environment are influenced by family socioeconomic status and cultural capital (Ishizawa, 2014; Moskal, 2014; Naik, 2014; Zavala et al., 2018). However, few studies have mentioned the relationship between family capital and sense of place. Similarly, study has shown that the perception of place is influenced by geospatial thinking (Tian et al., 2021). Family parenting style has also been shown to be an important influence on children’s spatial thinking (Borriello and Liben, 2018; Clingan-Siverly et al., 2021). However, few studies have focused on the mediating role of geospatial thinking between family capital and sense of place and on the influence of family capital on geospatial thinking.

Thus, the purpose of this study was to verify the relationship between family capital and sense of place as well as the mediating effect of geospatial thinking on this relationship. Understanding the relationship between these three variables is conducive to exploring the influencing factors and the inner mechanisms of sense of place. As well, two frequently reported factors that may significantly influence sense of place—gender and residential address (urban and suburban)—were considered as covariates and controlled in the process. In the following section, the definition of the three constructs, their affecting variables, and the relations between them is presented.

## Theoretical basis and hypothesis

### Family capital

*Family capital*, which is derived from social capital theory, refers to family income, education, occupation, and social relationships, and it represents the sum of various types of resources that a family possesses. Bourdieu believed that the forms of capital include social capital, economic, cultural, linguistic, and technological capital (Bourdieu, 1984, 1986; Johnson and Bourdieu, 1993). Coleman (1990) identified three types of family capital—human, financial, and social. Family economic status and the resources and wealth available to the family are defined as financial capital; human capital refers to the cognitive environment provided for children that can facilitate their learning and is usually expressed by the parents’ educational attainment; and social capital refers to the resources in the family’s interpersonal relationships that can facilitate children’s development.

Among the available studies, family socioeconomic status (SES), which is measured by the three indicators of parental education, occupational prestige, and income, reflects the family’s economic and human capital (Baker, 2014; Chan et al., 2018). Conger and Donnellan’s (2007) family investment model theory and other empirical study suggest that individuals’ behavioral, emotional, cognitive, and health status are influenced by family SES (Ackerman et al., 2004). Moreover, these effects begin before birth and continue into adulthood. In the twentieth century, numerous studies demonstrated that children with low SES are more likely to develop psychiatric disorders and symptoms of social maladjustment (Bolger et al., 1995; Lahey et al., 1995; Brooks-Gunn and Duncan, 1997; McCoy et al., 1999). In contrast, higher SES predicts better social cognition, higher independence, and lower aggression in preschoolers (Xie et al., 2020). Second, studies show that children living in poverty have limited access to resources for play and physical activity compared to children from higher-income families (Romero et al., 2001; Tandon et al., 2012), but it has also been noted that low-income families are more inclined to encourage their children to take advantage of their surroundings, while wealthier families are more concerned with opportunities for organized activity (Cottrell et al., 2015). In addition, family socioeconomic status is positively associated with health, and lower SES may lead to a higher risk of physical and mental health problems (Ji et al., 2020). A study shows that high levels of family support are positively associated with children’s wellbeing (Moscardino et al., 2021). Choi et al. (2019) found that the risk of suicide is higher for low-income groups than for high-income groups. In general, there is an impact on socioeconomic status on the medical conditions that people experience (Chan et al., 2018). For example, a study confirmed that patients with lower SES were more likely to suffer from ocular trauma (Kousiouris et al., 2022).

In daily life, families with higher SES have more resources to help with personal development (Matthews and Gallo, 2011; Frewen et al., 2015; Wang and Huang, 2021). For example, families with high levels of cultural capital are more likely to pay for remedial education for their children (Southgate, 2013). At the same time, several researchers have demonstrated that students’ happiness, health, and satisfaction with life are influenced by family capital (Novak et al., 2018; Kühner et al., 2021; Addae and Kühner, 2022). Conversely, limited family capital can be a barrier to children’s development (Chase-Lansdale et al., 2019; Ostroot and Backstrom, 2021).

Notably, researchers have paid particular attention to the impact of family capital on education (Sáenz et al., 2018; Guan and Ploner, 2020; Wang and Huang, 2021; Ren et al., 2022). Li and Qiu (2018) proposed two pathways through which family influences children’s academic performance: Parents compete for high-quality educational opportunities, and they change children’s study habits through their parenting behaviors and educational support. Gao et al. (2015) showed that factors such as family capital, place of origin, and place of birth significantly affect college students’ school performance. Another study confirmed the significant relationship between family social capital and students’ reading, math, and science abilities (Lan, 2013).

Various indicators have been used to measure family capital. One of the most common expressions of family capital is SES, which is represented by parents’ education, occupation, and income (Warner et al., 1949; Blau and Duncan, 1967; Haller and Portes, 1973; Buchmann, 2002). For instance, the Family Affluence Scale was designed by Currie et al. (1997) as part of the World Health Organization’s School Children’s Health Behavior in School-Aged Children research project. The survey of home education resources, part of the Trends in International Mathematics and Science Study, includes dictionaries, child-specific desks, computers, and number of books (Third International Mathematics and Science Study, 2019). The Programme for International Student Assessment (PISA) is the largest scale and most influential international education monitoring and evaluation project. Its student questionnaire (Programme for International Student Assessment, 2018) collects information about parent education level, parent occupation, family possessions, and the number of books in the home to analyze the respondent’s family environment. The *Family Capital Questionnaire* used in this study was adapted from this questionnaire.

### Sense of place

In the 1970s, Tuan and Lowenthal introduced the concept of sense of place (Tuan, 1974, 1975; Relph, 1976; Lowenthal, 1979), arguing that a *sense of place* includes both the inherent characteristics of a place, and the complex connections people have with it. This connection is reflected at the cognitive, behavioral, and emotional levels (Nelson et al., 2020). Since sense of place is a multidimensional concept (Steele, 1981; Stedman, 2002; Hashemnezhad et al., 2013), concepts such as place attachment, place identity, place dependency (Qian et al., 2011; Tapsuwan et al., 2011; Kudryavtsev, 2013), satisfaction (Stedman, 2003; Billig, 2005), community feeling, environment, and health (Williams et al., 2010; Soini et al., 2012) can be considered subordinate concepts of sense of place (Shamai, 1991).

Sense of place is a combination of environment and perception (Tuan, 1975; Smith and Relph, 1978; Brandenburg and Carroll, 1995; Mason and Sack, 1999). Therefore, the formation of a sense of place needs to consider not only the specific location and geographic context, but also the perception of the environment (Massey, 2008). Scholars have argued that sense of place is derived from lived experience and knowledge and is influenced by the external environment (Pred, 1983; Relph, 1997). For example, Soini et al. (2012) believed that sense of place is closely related to the experience of place. Other scholars argue that sense of place and emotion are inseparable (Lanouette, 2022). It has also been shown that urban environmental education is important in developing a sense of place among young people which can make them aware of the ecological value of urban landscapes and thus further promoting awareness of the benefits of protecting and managing the natural environment in cities (Kudryavtsev et al., 2012). In recent years, research in neuroscience has shown that behavioral, physical, perceptual, and emotional elements are all related to the formation of sense of place (Lengen and Kistemann, 2012; Campelo, 2015; McCunn and Gifford, 2021; Wells, 2021), which further demonstrates that sense of place is a combination of environment and perception.

In general, sense of place is influenced by many factors. Personal factors including demographic factors such as residential address (urban and suburban), gender, age, education, and length of residence are included (Hutson et al., 2019; Collins-Kreiner, 2020; Leather and Thorsteinsson, 2021). Environmental factors are related to the physical or social environment. The physical environment generally refers to the unique local characteristics of the place, including physical geography, history and culture, infrastructure and services, and architectural style (Stedman, 2002; Ortiz et al., 2004; Ali, 2019; Dea and Kusuma, 2021). Mohammadi (2021) showed that physical characteristics of urban spaces can affect sense of place by affecting human perception. Factors related to social environment include SES, social ties, holiday celebrations, religion, and welfare (De Bres and Davis, 2001; Mazumdar and Mazumdar, 2004; Williams and Kitchen, 2012).

Quantitative, qualitative, and mixed research methods have been used in the study of sense of place (Shamai, 1991; Jorgensen and Stedman, 2001; Shamsuddin and Ujang, 2008; Amsden et al., 2010; Vannini and Taggart, 2013). Frequently used quantitative methods are constructing models and developing scales. In terms of model construction, Relph (1976) interpreted sense of place factors as a stable natural environment, human activities, meaning, and place spirit. In terms of scale design, the classic local attachment scale was developed by Williams et al. (1992). In addition, Jorgensen and Stedman (2001), who divided place into three dimensions: place attachment, place dependence, and place identity, designed a 12-item scale for sense of place, which we adapted for this study.

Research has shown that a sense of place involves the everyday world and is built-up over both years of residence and involvement in the community (Tuan, 1974, 1975). A study showed that relationships with friends and family, relationships with special places, and length of residence have the most significant impact on sense of place (Hay, 1988), which shows that family and community have an important influence on the sense of place. Most studies have focused on the impact of community context on the sense of place from a meso perspective (Tester et al., 2011; McCunn and Gifford, 2021) and the relationship between community activities and sense of place (Gatti and Procentese, 2021). Zhang et al. (2020) found that urban riverfront landscapes play an important role in promoting residents’ sense of place. It has also been shown that students’ sense of place is effectively enhanced through participation in community activities (Kim et al., 2020).

At the family level in a micro perspective, the results of one study suggest that sense of place can be transmitted to children through their parents (Hay, 1998). For the individual, the family is a specific environment with unique material conditions and spiritual and cultural atmosphere conditions (Balda et al., 2019; Leto et al., 2019). Family capital can influence individuals’ physical and mental health, cognitive development, educational achievement, and future development, and it can influence individuals’ perceptions and behaviors. However, few studies have investigated the relationship between family capital and sense of place. After considering the influence of family capital on individuals, we derive the following hypothesis:

Hypothesis 1: Family capital has a positive predictive effect on sense of place.

### Geospatial thinking

For a long time, fields such as cognitive psychology and cognitive neuroscience have focused on the study of thinking and cognition (Holyoak and Spellman, 1993). Thinking, which is based on the perception but transcends its boundaries, is an advanced stage of understanding objective things that evolves with age and experience (Pyle, 1917). *Spatial thinking*, which began with psychological research on spatial cognitive abilities, is the essential and regular understanding of the spatial characteristics of geographic things, phenomena, and laws (Shepard and Metzler, 1971; Bethell-Fox and Shepard, 1988; Carroll, 1993). The definition of spatial thinking is still debated (McGee, 1979; Caplan et al., 1985; Linn and Petersen, 1985; Hegarty and Waller, 2004; Newcombe and Shipley, 2015). The Committee on Support for Thinking Spatially (2006) explained it as “a collection of cognitive skills consisting of spatial properties and concepts, the use of tools for representing spatial information, and tools for spatial reasoning processes” (p. 12). The concept of spatial thinking has attracted attention both in daily life and in education (Schultz et al., 2008; Bednarz and Lee, 2011; Zwartjes et al., 2017; Gagnier et al., 2022).

Geospatial thinking, a kind of spatial thinking specifically for the earth, landscape, and environment, is the basis for people’s cognition and understanding of the environment and of space (Gersmehl and Gersmehl, 2007; Bodzin et al., 2014). Bednarz (2011) defined it as the “knowledge, skills, and habits of mind that use representational tools such as concepts, spaces, maps or graphs, and reasoning processes to organize and solve problems.” In the beginning, instruments for testing spatial ability, developed by psychologists, provided instrumental support for the measurement of geospatial thinking. However, geographic researchers found that the use of psychological testing methods could cause errors in the assessment of geospatial thinking. This may be due to the fact that people think and reason differently about geographic (large scale) and manipulable (small scale) spaces and that graphical maps to some extent misrepresent the geographic spaces that they show (Mark and Freundschuh, 1995; Lee and Bednarz, 2009; Bednarz and Lee, 2019). Therefore, the development of appropriate methods for measuring geospatial thinking became an important task for geographic researchers. Kali et al. (1997) were the first to add assessment elements that fit the earth sciences to relevant tests. Current research primarily uses the spatial thinking ability test (STAT) developed and designed by Lee and Bednarz (2009), which includes seven question items on map layer overlay that evaluate factors such as selecting an appropriate address, reading topographic maps, locating maps based on verbal descriptions, identifying spatially relevant phenomena, creating contour maps, and distinguishing between types of spatial data (Lee and Bednarz, 2009; Bednarz and Lee, 2019). The *Geospatial Thinking Test* used in this study is adapted from this test.

Several studies have investigated the factors that are related to geospatial thinking (McGee, 1979; Golledge and Stimson, 1997; Gibson, 2014). One discipline related to geospatial thinking is neuroscience, and studies have confirmed the existence of regions of the brain dedicated to different types of spatial thinking (Gersmehl and Gersmehl, 2006, 2007, 2011). Also, neurological factors such as experience, genetics, and hormones are thought to be the source of individual differences in performance on tests of spatial thinking (McGee, 1979). Studies have also confirmed the positive effects on children’s spatial thinking development of the use of spatial language and gestures in parent–child interactions (Pruden et al., 2011; Casasola et al., 2020; Clingan-Siverly et al., 2021). As well, personal characteristics and experiences such as age, gender, education, spatial cognition, and home environment are main influencing factors for geospatial thinking (Golledge and Stimson, 1997; Gibson, 2014; Erskine et al., 2020). Studies showed that thinking is always embedded in a specific historical and cultural context and influenced by the resources available in the environment (Gauvain, 1995; Cole, 1998).

Furthermore, the role of education is particularly important. One study found that students engaged in geographic studies had better spatial thinking skills and abilities (Bednarz and Lee, 2019). Teaching equipment and information-technology tools are key to developing geospatial thinking skills (Lee and Bednarz, 2009; Kim and Bednarz, 2013; Ishikawa, 2016; Jo et al., 2016; Xiang and Liu, 2019), and schools in developed areas have relatively more funding to acquire such resources. In summary, the factors influencing geospatial thinking are somewhat related to the family, which leads to the following hypothesis:

Hypothesis 2: Family capital is a positive predictor of geospatial thinking.

Many studies reported that geospatial thinking was the basis for an individual’s perception and understanding of space and the environment (Gersmehl and Gersmehl, 2007; Kerski, 2008; Bodzin et al., 2014). Researcher has shown that specific structures in the human brain are dedicated to processing spatial information (Lengen and Kistemann, 2012). Another study has shown that people with higher spatial-literacy skills form better mental maps of place (Bednarz and Bednarz, 2008). Thus, an individual’s way of thinking, consciousness, life processes, social status, and health and wellbeing can also be influenced by a sense of place (Williams, 1998). Thus, we hypothesize as follows:

Hypothesis 3: Geospatial thinking has a positive predictive effect on sense of place.

Based on the literature and the three hypotheses above, we also propose the following hypothesis:

Hypothesis 4: Geospatial thinking mediates and buffers between family capital and sense of place.

**FIGURE 1:**
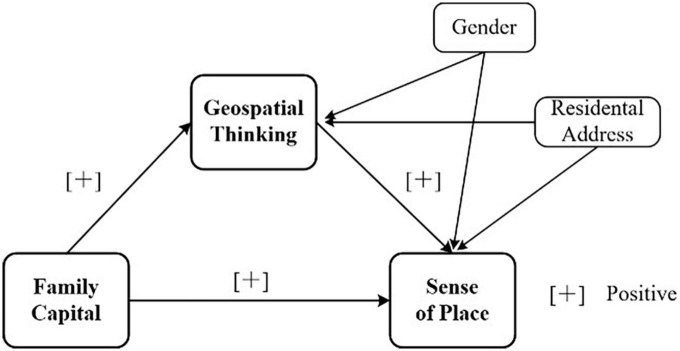
Relationships examined in the study.

Figure 1 shows a diagram of the mediation model proposed in the four hypotheses that depicts the relationships between the independent, mediator, and dependent variables and two covariates.

## Materials and methods

### Participants and procedures

To develop ideas and hypotheses, we conducted an exploratory focus-group interview in one school before the study design was finalized. Most of the interviewees indicated that they had little knowledge of the outside world and only had a keen sense of the place where they lived. A few of them mentioned that they had different feelings about various places as the result of family travel.

Data collection was conducted in public upper-secondary schools in western China. A total of 1,208 students aged 16–18 completed the survey questionnaire between 10 November and 30 November 2021. Before the students filled out the questionnaires, we explained the study to their parents, head teachers, and geography teachers, and consent was obtained from the students and their parents. During a break between classes, we distributed paper questionnaires to students. We collected the questionnaires after students completed them, and the resulting data were entered into the computer for analysis. After removing any incomplete responses, the number of valid questionnaires was 1,004.

### Materials

The questionnaire used to collect data for this study consisted of four sections: demographic information, the *Family Capital Questionnair*e, the *Geospatial Thinking Test*, and the *Sense of Place Scale.* The section on demographic information included gender and residential address. The questionnaires and scales used were adapted from their English-language versions, and we used the back-translation method (Brislin, 1970) to improve the quality of the translation: That is, one researcher translated the instrument from English to Chinese, then another researcher translated the Chinese version to English, and finally, a third researcher compared the three versions (original, translated, and back-translated) of the instrument for consistency between the original English and the translated text to avoid research error caused by translation errors.

#### Family capital questionnaire

Developed by Programme for International Student Assessment (2018), the *Family Capital Questionnaire* includes three dimensions: parents’ education level, parents’ occupation, and family belongings. Parental education is scored on a scale ranging from one (completion of primary education) to seven (completion of doctoral education). Parental occupation ranges from 1 (government/authority cadre/civil servant) to 12 (other unclassifiable occupations). Family economic status is determined by the number of items owned, with the corresponding number of points awarded and no points awarded for not owning items. The standardized *z* values of these six variables were included in the factor analysis based on available studies (Chung et al., 2017). We calculate the total score of household capital by principal component analysis, with higher scores reflecting higher levels of family capital.

#### Geospatial thinking test

Developed by Lee and Bednarz (2009), the *Geospatial Thinking Test* includes seven dimensions: map layer overlay, evaluating several factors to select an appropriate address, reading topographic maps, locating maps based on verbal descriptions, identifying spatially relevant phenomena, creating contour maps, and distinguishing between spatial data types. It consists of 16 items, such as “If you look along the arrows in 15, which picture in Figure 16 is closest to the landform you see?” “Real-world objects can be represented by points, lines (arcs) and faces (polygons). Please classify spatial data such as urban weather stations, the Yangtze River and its watershed, and the bus route of a primary school.” Students’ geospatial thinking is scored as one point for a correct answer and no points for a wrong answer. The higher the score, the higher the level of geospatial thinking of the participating students. In this study, the Cronbach’s alpha for the scale was 0.695.

#### Sense of place scale

Adapted from Jorgensen and Stedman (2001), the *Sense of Place Scale* includes three dimensions: place dependence, place attachment, and place identity. It consists of 12 questions, for example, “This place is relevant to me, a reflection of my existence” and “This place is my favorite place.” After discussion, some of the item statements were modified to accommodate the language habits and life experiences of students at the secondary-school level. The scale assesses respondents’ perceptions and feelings about place on a five-point Likert scale (1 = strongly disagree, 5 = strongly agree). An average score is calculated, and the higher the score, the stronger the sense of place. In this study, the internal consistency coefficient of the scale was 0.688.

### Data analysis

SPSS (version 26.0) and the PROCESS plug-in (version 4.0; Hayes, 2021) were used to analyze the data. First, we performed Harman’s single factor test to examine common method bias and ensure the validity of the data analysis (Podsakoff et al., 2003). All items in the questionnaire related to the three variables were tested. The results of unrotated principal component analysis showed that 11 factors had eigenvalues greater than 1, of which the contribution to the total variance was 56.162%. The first factor accounted for only 9.117%, which is far below the critical criterion of 40% (Zhou and Long, 2004), indicating that there was no significant common method bias. In other words, the variation between the independent and dependent variables was caused more by difference in the variables than by the methods of data collection and measurement. Following the test of common method bias, descriptive statistical analysis was performed: The mean and standard deviation of each variable were calculated to observe the trend of concentration and dispersion. Then, the Pearson correlation coefficients among the variables were calculated to test the closeness and variation patterns on all variables. Finally, a mediation analysis was conducted using the PROCESS plug-in in SPSS to explore the mediating role of geospatial thinking and further validate the four hypotheses of this study.

## Results

### Descriptive statistic and correlation analyses

Among the interviewees, 252 (25.10%) were male students and 752 (74.90%) were female students. As for the residential address, 600 students (59.76%) lived in urban areas and 404 (40.24%) in suburban areas. The results of the descriptive analysis of family capital, sense of place, and geospatial thinking are summarized in Table 1.

**TABLE 1:** Descriptive statistics for the three variables.

Variable	N	M	SD
Family capital	1,004	0.0185	1.7812
**Gender**			
Male	252	0.1458	1.9182
Female	752	−0.0241	1.7321
**Residential address**			
Urban	600	0.7876	1.7025
Suburban	404	−1.1237	1.1828
Sense of place	1,004	3.3735	0.4520
**Gender**			
Male	252	3.3401	0.5021
Female	752	3.3850	0.4338
**Residential Address**			
Urban	600	3.3890	0.4819
Suburban	404	3.3509	0.4032
Geospatial thinking	1,004	8.4900	2.6780
**Gender**			
Male	252	8.5400	2.9740
Female	752	8.4700	2.5730
**Residential Address**			
Urban	600	8.8600	2.7320
Suburban	404	7.9400	2.4980

Pearson’s product-moment correlation coefficients were computed to assess the relations among the variables. The results showed (see Table 2) a positive correlation between all three variables. First, family capital had a positive impact on sense of place, with a significant correlation (*r* = 0.204, *p* = 0.000). Second, family capital had a moderate positive impact on geospatial thinking, with a significant correlation (*r* = 0.351, *p* = 0.000). The positive correlation between geospatial thinking and sense of place (*r* = 0.238, *p* = 0.000) was also significant. That is, there was a significant positive relationship between family capital, geospatial thinking, and sense of place in this study.

**TABLE 2:** Pearson’s *r* for the three variables.

Variables	Family capital	Sense of place	Geospatial thinking
Family capital	1		
Sense of place	0.204**	1	
Geospatial thinking	0.351**	0.238**	1

**p < 0.01.

### Mediation analysis

To examine the mediating role of geospatial thinking in the relationship between family capital and sense of place, the PROCESS plug-in (version 4.0; Hayes, 2021) was used to perform the mediation analysis with family capital as the independent variable, sense of place as the dependent variable, and geospatial thinking as the mediating variable (Model 4). In accordance with the results of the literature review, gender and residential address were used as control variables. Therefore, students’ gender (male and female) and residential address (urban and suburban) were transformed into dummy variables before they were entered in the mediation model.

The results (see Table 3) showed that family capital has a significant positive predictive effect on sense of place (β = 0.0646, *t* = 7.0192, *p* < 0.001), and the prediction remains significant even when geospatial thinking is entered (β = 0.0474, *t* = 4.9666, *p* < 0.001). In addition, family capital is a significant positive predictor of geospatial thinking (β = 0.5451, *t* = 10.4027, *p* < 0.001). Also, geospatial thinking has a significant positive predictive effect on sense of place (β = 0.0317, *t* = 5.7878, *p* < 0.001). Subsequently, both the direct effect of family capital on sense of place and the mediating effect of geospatial thinking had bootstrap confidence intervals (95%) with no zero between their lower and upper limits (see Table 4). This suggests that, after controlling for gender and residential address variables, family capital can directly predict sense of place and predict it indirectly through geospatial thinking. The direct effect (0.04738) and the mediation effect (0.01725) accounted for 73.310 and 26.690% of the total effect, respectively. That is, family capital is a significant positive predictor of both sense of place and geospatial thinking, and geospatial thinking partially mediates the relationship between family capital and sense of place. Our findings about the mediating role of geospatial thinking may be only partial, demanding further attention.

**TABLE 3:** Results of mediation analysis for the observed variables.

Regression equation		Fitting indices				Significance
		
Outcome variable	Predictor variables	*R*	*R* ^2^	*F* (df)	β	*t*
Geospatial thinking		0.3518	0.1238	47.0902***		
	Gender				0.0308	0.1685
	Residential address				0.1140	0.5995
	Family capital				0.5451	10.4027***
Sense of place		0.2849	0.0812	22.0676***		
	Gender				0.0543	1.7156
	Residential address				0.0815	2.4799*
	Geospatial thinking				0.0317	5.7878***
	Family capital				0.0474	4.9666***
Sense of place		0.2244	0.0504	17.6824***		
	Gender				0.0552	1.7188
	Residential address				0.0851	2.5490*
	Family capital				0.0646	7.0192***

***p < 0.001, *p < 0.05.

**TABLE 4:** Total effect, direct effect, and indirect effect among the variables.

Effect	Effect Size	Boot SE	Boot LLCI	Boot ULCI	Relative effect size
Total effect	0.06463	0.0092	0.0466	0.0827	
Direct effect	0.04738	0.0095	0.0287	0.0661	73.310%
Indirect effect	0.01725	0.0036	0.0106	0.0248	26.690%

**FIGURE 2:**
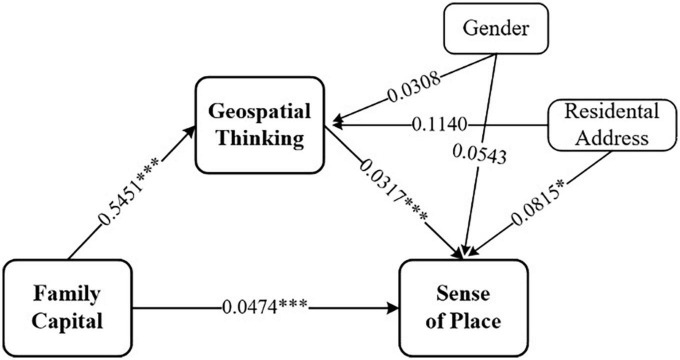
Mediation model showing relationships between family capital and sense of place and the mediating role of geospatial thinking. ***p < 0.001, *p < 0.05.

As shown in Table 3, when the association between family capital and sense of place was examined, residential address has an impact on sense of place, while the correlation between gender and sense of place was not significant. There was a significant effect of residential address on the level of sense of place (β = 0.0851, *t* = 2.5490, *p* < 0.05). Moreover, the effects of residential address on sense of place remained even when geospatial thinking was incorporated into the model. Residential address has a significant effect (β = 0.0815, *t* = 2.4799, *p* < 0.05), and the association between gender and geospatial thinking was not significant. We found that students from suburban areas have a higher level of geospatial thinking and sense of place. Figure 2 provides a graphic representation of these relationships.

## Discussion

### Discussion of the results

In this study, we created a mediation model that indicates the relationship between family capital and sense of place, as well as the mediating role of geospatial thinking. The results of this study are congruent with the hypotheses proposed and with previous research.

First, these results agree with Hypothesis 1: Family capital and sense of place were positively correlated. This finding implies that positive environment (Zhang et al., 2020) and positive perceptions and cognitions (Khan et al., 2020) have a facilitative effect on sense of place. Sense of place is the result of the interaction between individual perceptions and the external environment (Relph, 1997, 2007). Therefore, both personal characteristics and external environment can be influential factors in sense of place (Steele, 1981; Kaltenborn, 1998; De Bres and Davis, 2001; Stedman, 2002; Ortiz et al., 2004; Williams and Kitchen, 2012; Eanes et al., 2018; Hu and Chen, 2018; Rast, 2018; Sheybani and Poursoleiman Amiri, 2018). The family, which plays an important role for the individual, both acts as the environment and leads to differences in other factors (Evans and English, 2002; Ackerman et al., 2004; Frewen et al., 2015; Wang and Huang, 2021). In general, individuals with superior family capital are more likely to have a positive emotional response to a given environment. As posited by Tester et al. (2011), the level of attachment to place is higher for those living in high-quality public housing. Conversely, people of low SES are often unable to integrate into and use public spaces, which limits their participation in civic life and diminishes their sense of place in the community (Trawalter et al., 2021). Therefore, researchers attach great importance to the key role that families play in the development of individuals’ perceptions, behavior, and thinking skills (Ogg and Anthony, 2020; Li et al., 2021; Luo and Gao, 2021; Martins et al., 2021).

Second, the results of this study support Hypothesis 2 that family capital has a positive predictive effect on geospatial thinking. Our findings are consistent with the results of similar studies that have demonstrated that superior SES and cultural capital have a positive effect on thinking development (Gauvain, 1995; Cole, 1998; Gearin et al., 2018; Manstead, 2018). This result suggests that family capital promotes the development and improvement of geospatial thinking skills. Studies showed that the use of spatial language and gestures in parent–child interactions has a positive impact on children’s spatial thinking development (Pruden et al., 2011; Casasola et al., 2020; Clingan-Siverly et al., 2021). Likewise, family capital influences factors such as educational attainment and home environment. Tomaszewski et al. (2015) found that urban students outperformed rural students on tests of geospatial thinking, and Johnson et al. (2022) demonstrated that children from higher-income families performed better in spatial. However, students from disadvantage have performed less well on spatial tasks (Carr et al., 2018). A possible explanation for this might be that improving students’ spatial thinking skills requires the support of various related activities and information technology (Pietsch and Jansen, 2012; Weckbacher and Okamoto, 2012; Xiang and Liu, 2019; Koc and Topu, 2022) and that more affluent or more cultured families are more able to provide for their children (Cottrell et al., 2015).

Third, our findings are in accord with Hypothesis 3 and those studies indicating a significant positive correlation between geospatial thinking and sense of place (Bednarz and Bednarz, 2008; Jepson and Sharpley, 2015). The possible explanation is that both geospatial thinking and sense of place are essentially related to neurological processes, and geospatial thinking is the basis for developing a sense of place (Schinazi and Thrash, 2018; Nicosia, 2019). As posited by Massey (2008), perception influences the formation of sense of place, and geospatial thinking is a tool for perceiving the environment. It has been confirmed that spatial thinking is involved in the formation and development of the sense of place (Hay, 1998; Johnson, 2007; Lengen and Kistemann, 2012). In other words, when interacting with the nearby environment, individuals use geospatial thinking to encode spatial information and thus develop a sense of place (Gifford, 2014; McCunn and Gifford, 2021).

Fourth, our findings are consistent with Hypothesis 4. We found that geospatial thinking mediates between family capital and sense of place, which revealed a pathway for family capital to act on sense of place. First, students with better family capital tend to have better geospatial thinking, which is related to familial influence on individual activities, on life experiences such as language, and on neurological factors such as perception and genetics (McGee, 1979; Ackerman et al., 2004; Cottrell et al., 2015; Frewen et al., 2015; Clingan-Siverly et al., 2021). In addition, geospatial thinking has been proven to be involved in the development of a sense of place. Using functional magnetic resonance imaging, researchers have described the topography of active cortical zones and subcortical formations in the human brain during spatial thought and found specialized structures for processing spatial information (Hölscher et al., 2003; Kentros et al., 2004; Ivanitskii et al., 2015; Kozlova et al., 2016). It has also been shown that the human brain can use spatial information to encode and interpret emotional reactions to meaningful places (McCunn and Gifford, 2018). Overall, the essence of geospatial thinking is a collection of spatial cognitive skills, and relevant research in neuroscience has demonstrated the facilitative effect of spatial cognition on sense of place (The Committee on Support for Thinking Spatially, 2006; Bednarz, 2011; Lengen and Kistemann, 2012). In other words, people with high levels of geospatial thinking in specific environments and activity contexts can more effectively stimulate relevant areas of the cerebral cortex to produce stronger perception and understanding of the outside world, thus positively influencing the sense of place.

Fifth, the results indicate that the effect of gender on geospatial thinking and sense of place is not significant. There are different perspectives in the study of the relationship between gender and geospatial thinking. Some scholars point out that there is no significant difference in geospatial thinking among the different gender, and this is because people have equal exposure to maps that contribute to the development of spatial thinking through smartphones and Internet mapping applications (Bednarz and Lee, 2011; Larianne, 2018). In contrast, others hold the opposite view, with boys outperforming girls in geospatial thinking (Miller and Halpern, 2014; Shin et al., 2016). A possible explanation for this is that androgens promote the ability to process spatial information, so that boys are better at spatial aspects than girls (Núñez et al., 2020). In addition, family socialization influences also play a significant role (Cicognani et al., 2014): Traditionally, male adolescents are more encouraged by parents to become autonomous and make different experiences outside the family than female adolescents, which improved their sense of community environment; women in adulthood, especially those who have kids, get more opportunities to experience the local environment, which helps improve their sense of community (Wood et al., 2013). Conversely, Scannell and Gifford (2010) found that men and women did not differ in their levels of civic or natural place attachment. Indeed, the unequal gender distribution of respondents may have influenced the results, but the large sample size ensures the reliability of the results of this study. The results also indicated that place of residence significantly influenced the level of sense of place, and students who lived in the suburbs had a higher level of sense of place, which is consistent with the results of other similar studies where place of residence was considered as a factor influencing individuals’ level of sense of place (Lim and Barton, 2010). For example, the natural environment of the suburbs can inspire stronger place attachment (Sanecka et al., 2020).

It is worth noting that in this study, geospatial thinking only partially mediates the relationship between family capital and sense of place. The mediation analysis showed that the mediation effect of geospatial thinking was 26.690%. In other words, when geospatial thinking skills are low, higher family capital is still likely to increase sense of place.

### Implications

In terms of theoretical implications, this study is unique in linking family capital to sense of place, which deepens the understanding of the impact of family capital on students’ sense of place. Furthermore, the mediating and buffering effects of geospatial thinking derived from this study suggest that family capital may enhance geospatial thinking skills and promote a sense of place. In terms of practical implications, the relationships between the three variables proposed in this study may help families, teachers, and other stakeholders gain a deeper understanding of the mechanisms that shape students’ sense of place and lay a foundation for them to better help students develop their sense of place.

### Limitations and future directions

This study is subject to certain limitations. First, our sample is not population-representative because of the sampling procedures used and, as a result, may not be representative of other regions and time periods assessed. Second, all the participants were from the same region, which may undermine the generalizability of the research findings. Third, the imbalance in the gender ratio of the participants may also hamper the generalization of the results. In future, researchers could use a longitudinal survey design to collect data over a period and recruit participants equally from different schools in different regions, focusing on the impact of city size on the level of geospatial thinking and sense of place, which allows for a deeper understanding of the development of spatial thinking and sense of place in different contexts of time. In addition, they could explore which dimension of geospatial thinking mediates the relationship between family capital and sense of place. Finally, by analyzing the mechanisms underlying the influence of family capital on sense of place and geospatial thinking, we provide a direction for future consideration of how to develop sense of place and geospatial thinking in individual students with a lower level of family capital.

## Conclusion

This study explored the relationship between family capital and sense of place and the role of geospatial thinking in mediating between the two. The results indicate that participating upper-secondary-school students with a higher level of family capital had a better sense of place. In addition, students with stronger geospatial thinking skills had a better sense of place than students with weaker geospatial thinking skills. Notably, most variance in sense of place was still attributable to family capital, although geospatial thinking did play a role.

## Data availability statement

The raw data supporting the conclusions of this article will be made available by the authors, without undue reservation.

## Ethics statement

The studies involving human participants were reviewed and approved by the Ethics Committee of Zhejiang Normal University. Written informed consent to participate in this study was provided by the participants’ legal guardian/next of kin.

## Author contributions

YX and JZ designed the research. JZ, XaL, TS, XnL, JG, and ZA carried out the literature search and data analysis. JZ, XaL, TS, XnL, JG, ZA, and YX wrote the manuscript. All authors have read and agreed to the submitted version of the manuscript.
